# Abscisic acid implicated in differential plant responses of *Phaseolus vulgaris* during endophytic colonization by *Metarhizium* and pathogenic colonization by *Fusarium*

**DOI:** 10.1038/s41598-021-90232-4

**Published:** 2021-05-31

**Authors:** Shasha Hu, Michael J. Bidochka

**Affiliations:** Department of Biological Sciences, Brock University, St. Catharines, ON L2S 3A1 Canada

**Keywords:** Fungal host response, Microbe, Plant hormones

## Abstract

*Metarhizium robertsii* is an insect pathogen as well as an endophyte, and can antagonize the phytopathogen, *Fusarium solani* during bean colonization. However, plant immune responses to endophytic colonization by *Metarhizium* are largely unknown. We applied comprehensive plant hormone analysis, transcriptional expression and stomatal size analysis in order to examine plant immune responses to colonization by *Metarhizium* and/or *Fusarium*. The total amount of abscisic acid (ABA) and ABA metabolites decreased significantly in bean leaves by plant roots colonized by *M. robertsii* and increased significantly with *F. solani* compared to the un-inoculated control bean plant. Concomitantly, in comparison to the un-inoculated bean, root colonization by *Metarhizium* resulted in increased stomatal size in leaves and reduced stomatal size with *Fusarium*. Meanwhile, expression of plant immunity genes was repressed by *Metarhizium* and, alternately, triggered by *Fusarium* compared to the un-inoculated plant. Furthermore, exogenous application of ABA resulted in reduction of bean root colonization by *Metarhizium* but increased colonization by *Fusarium* compared to the control without ABA application. Our study suggested that ABA plays a central role in differential responses to endophytic colonization by *Metarhizium* and pathogenic colonization by *Fusarium* and, we also observed concomitant differences in stomatal size and expression of plant immunity genes.

## Introduction

*Metarhizium robertsii* is an entomopathogenic fungus but it can also colonize the plant root rhizoplane and grow endophytically between or within cortical root cells^1^. This symbiotic interaction offers benefits to the plant by improving plant growth, antagonizing plant pathogens and herbivores, and enhancing plant tolerance to abiotic stresses^2^. This fungus is able to translocate nitrogen directly from infected insect cadavers to the plant host in exchange for plant photosynthate^3^. *M. robertsii* also confers protection against root rot of haricot bean caused by *Fusarium solani* f. sp. *phaseoli*^4^. *F. solani* can cause up to 84% yield loss in bean due to root rot when left unmitigated^5^.

There are few studies on plant immune responses to endophytic colonization by *Metarhizium*. For example, root colonization by *M. anisopliae* in peanuts triggered differential expression of genes involved in ethylene responsive transcription factors, dehydration-responsive element-binding proteins, nitrate transporters, and transcription factors^6^. Plant hormones play bifunctional roles in regulating plant growth and defense. The salicylic acid (SA) response pathway is activated by biotrophic pathogen attack, and jasmonic acid (JA) and ethylene coordinate defense responses against necrotrophic pathogens and herbivores^7^. *M. anisopliae* can decrease the concentration of SA in peanut roots compared to the un-inoculated control, which indicated suppression of plant defense during endophytic colonization^6^. However, increased levels of SA and JA in maize root was reported during endophytic colonization by *M. anisopliae*^8^. Additionally, exposure of *M. robertsii* to SA in vitro resulted in conidial thermotolerance but with decreased conidial yield^9^. Reduced abscisic acid (ABA) and elevated JA levels were observed in *Metarhizium*-inoculated soybeans compared to the un-inoculated control under salinity stress, which suggests decreased stress in *M. anisopliae* inoculated-plants^10^.

ABA is a major plant hormone, which plays a central role in plant physiological processes, as well as responses to abiotic and biotic environmental stresses. It is involved in different stages of plant growth, such as seed germination, leaf senescence, and bud dormancy^11^. Adaptation to drought, low temperature and salt stress is regulated by ABA signaling pathways in plants^11^. An increase in ABA concentration promotes the closure of stomata that in turn minimizes transpirational water loss^12^. ABA can also mediate infection by regulating the size of the stomatal aperture which is an entry portal for pathogens^13^.

ABA plays complex roles during plant–microbe interactions. ABA usually exerts a negative effect on plant resistance to pathogens by suppressing host immune responses, such as with *Botrytis cinerea* in tomato^14^, *Ralstonia solanacearum* in tobacco^15^, and *Magnaporthe oryzae* in barley^16^. However, ABA can increase resistance of *Arabidopsis* to the necrotrophic fungus, *Alternaria brassicicola*^17^. In mutualistic plant–microbe interactions, ABA can promote colonization and establishment of compatible interactions with arbuscular mycorrhizal fungus *Glomus intraradices* in tomato^18^. During root nodule symbiosis, ABA acts as a negative regulator by inhibiting root nodule formation by rhizobia after exogenous ABA application^19^.

Here we investigated immune responses by the bean plant in response to endophytic colonization by *M. robertsii* and pathogenic colonization by *F. solani*. The influence of ABA on stomatal size, expression of ABA catabolic genes, and immune response genes was assessed during endophytic (*Metarhizium*) and pathogenic (*Fusarium*) colonization as well as co-colonization by *Metarhizium* and *Fusarium*. Our findings suggest that ABA is implicated in modulating diverse plant-pathogen and plant-endophyte interactions.

## Results

### Quantification of bean hormones in foliar tissues

Comprehensive hormone profiling of 39 plant hormones were examined in 14 days bean foliar tissues, including abscisic acid and abscisic acid metabolites, auxins, cytokinins, gibberellins and 1-aminocyclopropane-1-carboxylic acid (Supplementary Table S1). The (*trans*) zeatin-O-glucoside and (*cis*) zeatin-O-glucoside showed a maximum amount of 20 ng g^−1^ DW (dry weight) with no significant differences in fungal colonized plant compared to the un-inoculated plant. The indole-3-acetic acid showed no significant differences among fungal treatment plant groups compared to the un-inoculated plant. The ethylene precursor, 1-aminocyclopropane-1-carboxylic acid, showed significantly greater amounts in bean colonized by *Metarhizium* compared to the un-inoculated control plant (Supplementary Fig. S1). The amount of tested gibberellins was low and showed no significant changes in infected samples (data not shown) compared to the un-inoculated plant.

**Figure 1:**
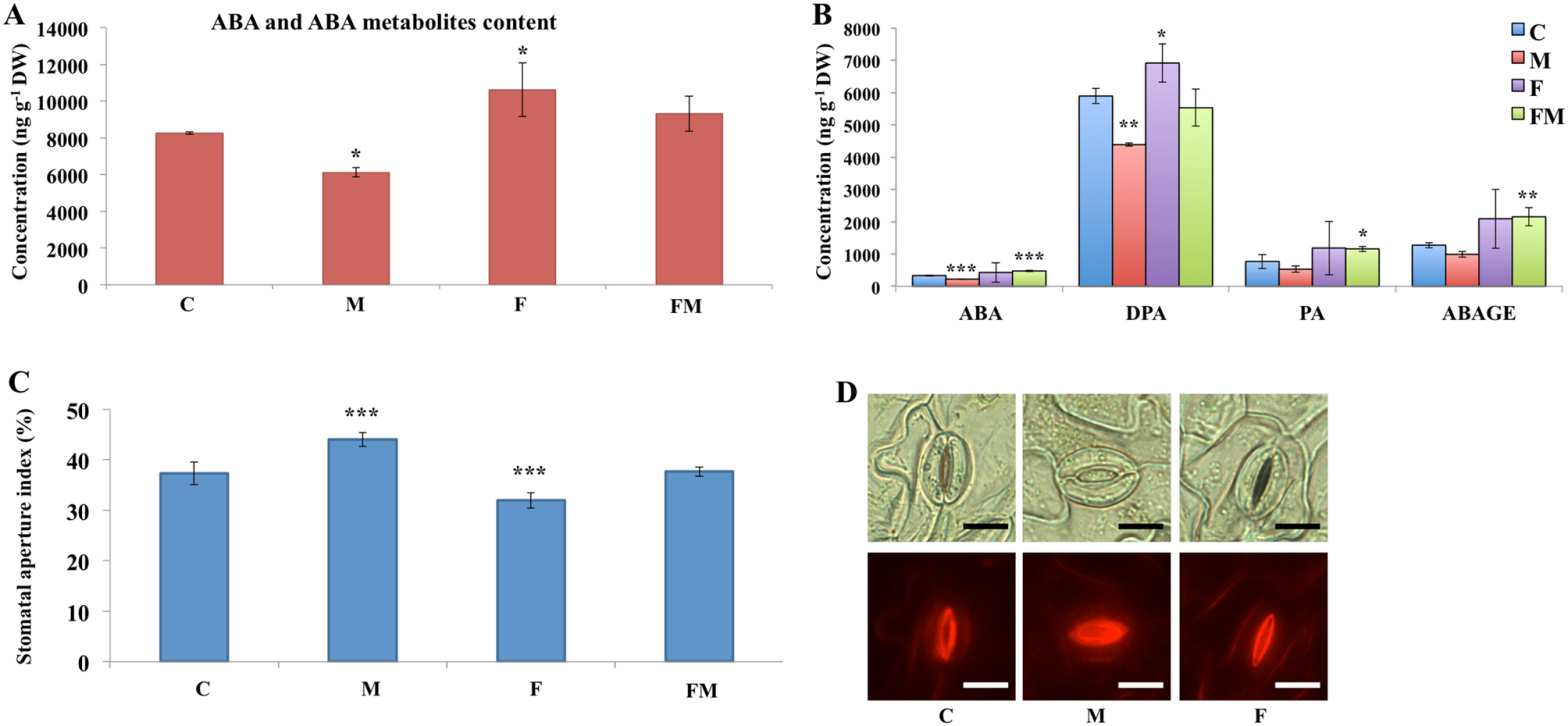
ABA and metabolites and stomatal apertures sizes in bean leaves. Bean leaves were collected at 14 days in un-inoculated control (C), plants colonized by *M. robertsii* (M), *F. solani* (F), or *M. robertsii* together with *F. solani* (FM). (**A**) Total amount of ABA metabolites in bean leaves of un-inoculated control and bean colonized by different fungi. (**B**) Details of ABA and ABA metabolites in bean leaves of un-inoculated control and bean colonized by different fungi. *cis*-Abscisic acid (ABA), Dihydrophaseic acid (DPA), Phaseic acid (PA), Abscisic acid glucose ester (ABAGE). (**C**) The stomatal aperture index in bean leaves after 14 days in un-inoculated control and bean colonized by different fungi. Standard deviations are shown. Data were analyzed by one-way ANOVA with Fisher's least-significant difference (LSD) test. Statistical differences shown; Asterisks indicate statistically significant differences relative to un-inoculated control, *P asterisks, **P < 0.01, ***P < 0.001*.* D, Light microscopy (upper panel) and fluorescence microscopy after staining with rhodamine 6G (lower panel) of stomatal apertures in bean under 250 × magnifications. Scale bar is 15 μm.

ABA and ABA metabolites were detected in relatively greater levels compared to other plant hormones. The amount of ABA and ABA metabolites showed significant differences between the un-inoculated bean leaves and the bean plant colonized by *Metarhizium* or/and *Fusarium* (Fig. 1A,B). The total amount of detected ABA and ABA metabolites decreased significantly in bean leaves by plants roots colonized by *Metarhizium* and increased significantly in bean plants colonized by *Fusarium*, compared to the un-inoculated bean plant (Fig. 1A). The amount of *cis*-abscisic acid (ABA) decreased significantly in the *Metarhizium* colonized bean leaves and increased significantly in those co-colonized by *Metarhizium* and *Fusarium* compared to the un-inoculated control bean plant (Fig. 1B). The main metabolite detected in the ABA metabolism pathway was dihydrophaseic acid (DPA), which showed significantly lower levels in bean colonized by *Metarhizium* and higher levels in bean colonized by *Fusarium* compared to the un-inoculated control. The amount of phaseic acid (PA) and abscisic acid glucose ester (ABAGE) increased significantly in bean co-colonized by *Metarhizium* and *Fusarium* compared to the un-inoculated bean plant (Fig. 1B).

### Analysis of stomatal aperture index (SAI)

An increase in the level of ABA can initiate a signaling cascade which results in a decrease in the size of the leaf stomatal aperture^12^. Based on the observation of decreased levels of ABA in bean leaves during endophytic colonization by *Metarhizium*, we tested the size of stomatal apertures in the bean plant colonized by different fungi, which was expressed as SAI^20^. The SAI significantly increased in bean leaves when the plant root was colonized by *Metarhizium* (*P* < 0.001) and significantly decreased in bean colonized by *Fusarium* (*P* < 0.001), compared to the leaves in the un-inoculated bean. These results were consistent with the decreased amount of total ABA metabolites amount in bean plants colonized by *Metarhizium* and increased amounts of ABA in *Fusarium* colonized plant (Fig. 1A,C,D). Colonization of bean root by *Metarhizium* resulted in decreased ABA in bean leaves, which is correlated with the opening of the stomatal aperture. While pathogenic colonization by *Fusarium* resulted in increased ABA and closure of the stomata in bean leaves.

### Root colonization of *Metarhizium* and *Fusarium* after exogenous ABA application

**Figure 2:**
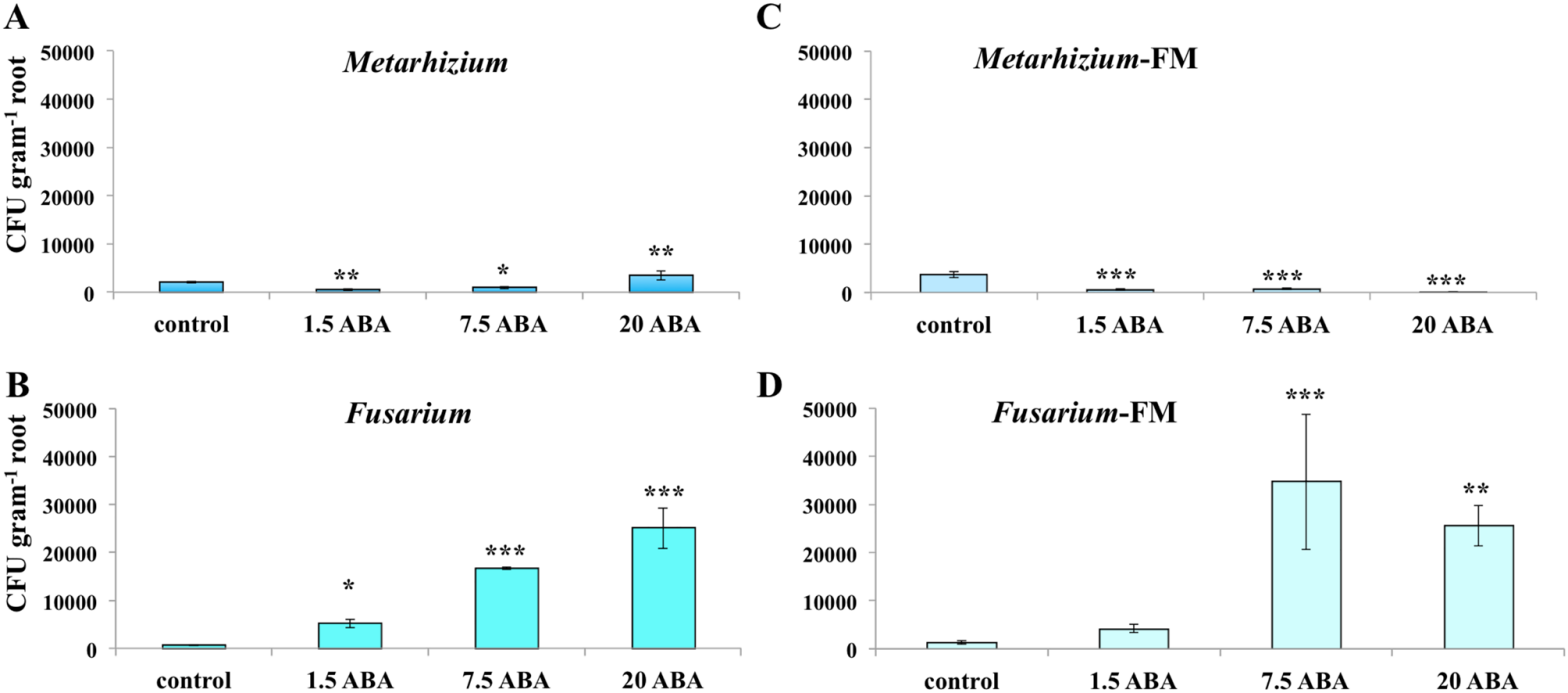
CFU of *M. robertsii* or *F. solani* in bean root homogenate after exogenous application of ABA. X-axis represents values of ABA μg per bean plant. FM: *F. solani* and *M. robertsii* treatment group. Standard error bars are shown. Data were analyzed by one-way ANOVA with Fisher's least-significant difference (LSD) test. Statistical differences shown; Asterisks indicate statistically significant differences relative to corresponding fungus colonized plant without ABA application *P < 0.05, **P < 0.01, ***P < 0.001.

After exogenous application of ABA, root colonization by fungi was determined by counting the colony-forming units (CFU) in root homogenates. All values are in CFU g^−1^ of bean root. Compared to the plant root colonized by *Metarhizium* without ABA application, the CFU g^−1^ root of *Metarhizium* decreased significantly after application of 1.5 μg ABA (*P* < 0.01) and 7.5 μg ABA (*P* < 0.05) per bean plant, and increased after application of 20 μg ABA (*P* < 0.01) per bean plant (Fig. 2A). However, application of ABA increased root colonization of *Fusarium* in the bean plant (Fig. 2B). In comparison to bean colonized by *Fusarium* without ABA application, root colonization of *Fusarium* increased significantly after application of 1.5 μg ABA (*P* < 0.05), 7.5 μg ABA (*P* < 0.001) and 20 μg ABA (*P* < 0.001), respectively. In the bean plant co-colonized by *Metarhizium* and *Fusarium*, a similar decrease in root colonization by *Metarhizium* and increased root colonization by *Fusarium* was observed after exogenous ABA application compared to the corresponding fungi colonized bean without ABA application (Fig. 2C,D). Specifically, compared to the plant co-colonized by *Metarhizium* and *Fusarium* without ABA application, root colonization of *Metarhizium* decreased significantly after application of 1.5 μg (*P* < 0.001), 7.5 μg (*P* < 0.001) and 20 μg ABA (*P* < 0.001) in the bean co-colonized by *Metarhizium* and *Fusarium* after ABA colonization (Fig. 2C). Meanwhile, after the application of 7.5 μg (*P* < 0.001) and 20 μg (*P* < 0.01) ABA, root colonization of *Fusarium* increased significantly in the bean co-colonized by *Metarhizium* and *Fusarium* compared to the corresponding fungi colonized bean plant without ABA application (Fig. 2D). Decreased root colonization of *Metarhizium* and increased root colonization of *Fusarium* after the exogenous application of 7.5 μg ABA indicated that ABA plays a negative role in root colonization by *Metarhizium* but improves root colonization by *Fusarium* in bean plant.

During ABA application in vitro, there were no differences in colony morphology and conidial production with *M. robertsii* and *F. solani* compared to the growth of fungi on PDA without ABA application (Supplementary Fig. S2). This result indicated that the differential root colonization of *M. robertsii* and *F. solani* during exogenous application of ABA, might not result from the direct effect of plant ABA on the fungi, and may instead be associated with the differential plant responses triggered by exogenous application of ABA such as differential expressions and/or post translation regulation of plant immunity genes.

### Expression of genes involved in ABA catabolism pathway

**Figure 3:**
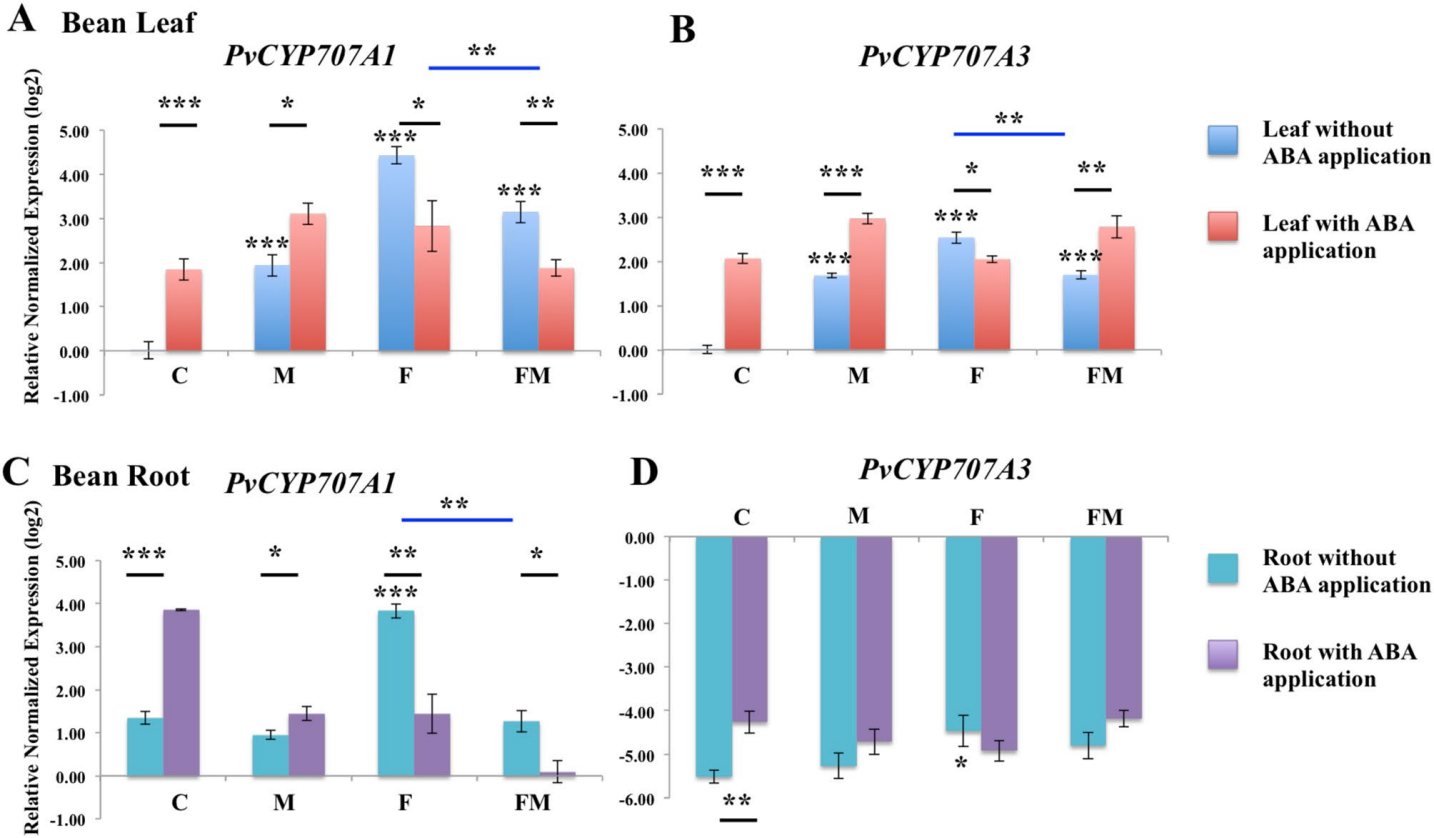
Relative expression of genes involved in the ABA catabolic pathway in bean leaves (**A**, **B**) and roots (**C**, **D**). RNA was extracted at 14 days bean leaves and root in un-inoculated control (C), colonized by *M. robertsii* (M), *F. solani* (F), and *M. robertsii* together with *F. solani* (FM). For each gene, the expression level in un-inoculated control bean leaf was set to 1. Standard errors are shown. Data were analyzed with standard *t*-test in Bio-Rad CFX Manager software. Statistical differences are shown; *P < 0.05, **P < 0.01, ***P < 0.001. Asterisk alone indicates significant differences in gene expression in the tested group when compared to that of the un-inoculated control. Asterisk above the black line indicates significant differences in gene expression in the plant with exogenous ABA application compared to the corresponding plant without ABA application. Asterisk above blue line indicates significant difference in gene expression in the plant co-colonized by *M. robertsii* and *F. solani* compared to that in the plant colonized only by *F. solani* or *M. robertsii*. ABA 8′-hydroxylase 707A1 (*PvCYP707A1*) and ABA 8’-hydroxylase 707A3 (*PvCYP707A3*). The experiment was repeated twice with similar results.

DPA is the end product of the ABA metabolic pathway through 8′-hydroxylation, and the high levels of DPA observed suggested that initially high levels of ABA were produced and subsequently metabolized. PvCYP707As are cytochrome P450 monooxygenases, which catalyze the hydroxylation of ABA at the 8’ position during the catabolism from ABA to DPA^21^. The expression levels of *PvCYP707A1* and *PvCYP707A3* were assessed in bean leaves and roots during endophytic colonization by *Metarhizium* and pathogenic colonization by *Fusarium* compared to the un-inoculated control bean plant. Figure 3A,B show relative transcript levels of *PvCYP707A1* and *PvCYP707A3* in bean leaves among different treatments of bean plants. The expression of *PvCYP707A1* and *PvCYP707A3* in bean leaves increased significantly in the plant treatments with different fungi, when compared to the un-inoculated control plant leaves. Specifically, plant treatment with *Metarhizium* resulted in significantly greater expression of *PvCYP707A1* (*P* < 0.001) and *PvCYP707A3* (*P* < 0.001) compared with the un-inoculated control bean leaves. Plant treatment with *Fusarium* showed increased expression for *PvCYP707A1* (*P* < 0.001) and *PvCYP707A3* (*P* < 0.001) when compared to the un-inoculated control bean leaves*.* Bean plant co-colonized by *Metarhizium* and *Fusarium* showed increased expression of *PvCYP707A1* (*P* < 0.001) and *PvCYP707A3* (*P* < 0.001) in comparison to the un-inoculated control bean leaves. Treatment with *Metarhizium* together with *Fusarium* significantly decreased the expression of *PvCYP707A1* (*P* < 0.01) and *PvCYP707A3* (*P* < 0.01) in bean leaves, compared to the plant infected only by *Fusarium* (Fig. 3A,B)*.* In bean roots, significantly increased expression of *PvCYP707A1* (*P* < 0.001) and *PvCYP707A3* (*P* < 0.05) was also observed in the plant colonized by *Fusarium*, compared to the expression of these two genes in un-inoculated control bean root (Fig. 3C,D). Meanwhile, the significantly decreased expression of *PvCYP707A1* (*P* < 0.001) was also found in the bean root treated with *Metarhizium* and *Fusarium*, compared to the bean root colonized only by *Fusarium* (Fig. 3C).

The ABA application experiment showed that exogenous ABA differentially affected root colonization by *Metarhizium* and *Fusarium*. Exogenously applied ABA (7.5 μg) was used to assess transcriptional expression of genes associated with ABA catabolic pathways in bean plant during fungal colonization. The differential changes in the expression of *PvCYP707A1* and *PvCYP707A3* were observed after exogenous ABA application in the plant colonized by *Metarhizium* and *Fusarium* (Fig. 3). In bean leaves and roots, the expression of *PvCYP707A1* was significantly up-regulated after exogenous ABA application in the un-inoculated control plant group (*P* < 0.001 for both leaves and roots) and bean plant colonized by *Metarhizium* (*P* < 0.05 for both leaves and roots) when compared to the corresponding plant without ABA application (Fig. 3A,C). While significant down-regulation of *PvCYP707A1* was observed in roots and leaves of bean colonized by *Fusarium* (*P* < 0.05 for leaves, *P* < 0.01 for roots) and bean plant co-colonized by *Metarhizium* and *Fusarium* (*P* < 0.01 for leaves, *P* < 0.05 for roots) after ABA application, in comparison to the corresponding fungi colonized plant without ABA application (Fig. 3A,C). For the expression of *PvCYP707A3* in bean leaves, significant up-regulation was observed after ABA application in un-inoculated control bean (*P* < 0.001), bean colonized by *Metarhizium* (*P* < 0.001) and bean co-colonized by *Metarhizium* and *Fusarium* (*P* < 0.01), relative to the corresponding bean plants without ABA application (Fig. 3B). Similar to the expression of *PvCYP707A1* in bean colonized by *Fusarium* during the ABA application, the expression of *PvCYP707A3* in bean leaves was significantly down-regulated in *Fusarium* colonized after ABA application (*P* < 0.05), compared to bean colonized by *Fusarium* without ABA application (Fig. 3B). The only significant difference of the expression of *PvCYP707A3* in bean roots after ABA application was found in the un-inoculated control bean plant, which showed significant up-regulation (*P* < 0.01) after ABA application compared to the un-inoculated plant without ABA application (Fig. 3D). Similar trends were observed in the expression of *PvCYP707A1* and *PvCYP707A3* in un-inoculated control plant and plant colonized by *Metarhizium* during the exogenous ABA application and indicated similar regulation of ABA catabolic genes during endophytic colonization by *M. robertsii* and un-inoculated control plant, which is different from that in the plant colonized by *Fusarium*. The up-regulation of *PvCYP707A1* and *PvCYP707A3* in plant colonized by *Metarhizium* and down-regulation of *PvCYP707A1* and *PvCYP707A3* in plant colonized by *Fusarium* after exogenous expression of ABA further confirm that ABA can work differentially on the expression of bean catabolic genes during colonization by these two fungi after exogenous application.

### Expression of genes associated with the immune response

**Figure 4:**
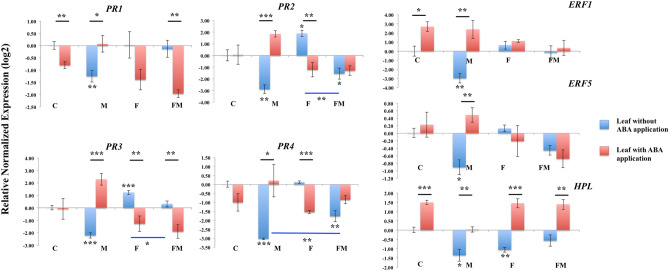
Relative expression of genes involved in plant immune responses in bean leaves. RNA was extracted at 14 days from bean leaves in un-inoculated control (C), colonized by *M. robertsii* (M), *F. solani* (F), and *M. robertsii* together with *F. solani* (FM). For each gene, the expression level in un-inoculated control bean leaf was set to 1. Standard errors are shown. Data were analyzed with standard *t*-test in Bio-Rad CFX Manager software. Statistical differences are shown; *P < 0.05; **P < 0.01; ***P < 0.001. Asterisk alone indicates significant difference in gene expression in the tested group when compared to that in the un-inoculated control. Asterisk above black line indicates significant differences in gene expression in the plant with exogenous ABA application compared to that in the corresponding plant without ABA application. Asterisk above blue line indicates significant difference in gene expression in the plant co-colonized by *M. robertsii* and *F. solani* compared to that in the plant colonized only by *F. solani* or *M. robertsii*. Pathogenesis related protein 1 (*PR1*), Beta 1–3 endoglucanase (*PR2*), Chitinase class I (*PR3*), Pathogenesis related protein 4 (*PR4*), Ethylene-Responsive Transcription Factor 1 (*ERF1*), Ethylene-Responsive Transcription factor 5 (*ERF5*), and Hydroperoxide lyase (*HPL*). The experiment was repeated twice with similar results.

To further examine differential immune responses of bean plant involved in the endophytic colonization by *Metarhizium* and pathogenic colonization by *Fusarium*, we tested the expression of immune genes, which were reportedly involved in immune responses associated with ABA^22^. The expression of immune response genes was significantly down-regulated in plants colonized by *Metarhizium* in comparison to the un-inoculated control bean (Fig. 4).

The expression of *PR1* (Pathogenesis related protein 1, XM_007154263), *PR2* (Beta 1–3 endoglucanase, XM_007154264), *PR3* (Chitinase class I, XM_007137247), *PR4* (Pathogenesis related protein 4, XM_007147114), *ERF1* (Ethylene-Responsive Transcription Factor 1, XM_007144028), *ERF5* (Ethylene-Responsive Transcription factor 5, XM_007157197), and *HPL* (Hydroperoxide lyase, XM_007149930) were significantly down-regulated in plants colonized by *Metarhizium* in comparison to the un-inoculated control plant. The expression of *HPL* (*P* < 0.01) was also significantly down-regulated in *Fusarium* colonized bean, compared to the un-inoculated control plant. However, in comparison to the un-inoculated control plant, *PR2* (*P* < 0.05) and *PR3* (*P* < 0.001) showed significantly up-regulated expression in plant colonized by *Fusarium*. When co-colonized by *Metarhizium* and *Fusarium*, *PR2* (*P* < 0.01), *PR3* (*P* < 0.05) and *PR4* (*P* < 0.01) showed significant different expression compared to the plant only infected by *Fusarium* or *Metarhizium*. *PR2*, *PR3*, and *PR4* may be involved in the interactions between *Metarhizium* and *Fusarium* in bean plant when bean is concomitantly colonized by both fungi^4^.

Changes in plant immune responses after ABA application were observed. In bean plant colonized by *Metarhizium*, a significant up-regulation was observed after ABA application in the following immune genes; *PR1* (*P* < 0.05), *PR2* (*P* < 0.001), *PR3* (*P* < 0.001), *PR4* (*P* < 0.05), *ERF1* (*P* < 0.01), *ERF5* (*P* < 0.01) and *HPL* (*P* < 0.01), compared to the plant colonized by *Metarhizium* without ABA application (Fig. 4). In the bean plant colonized by *Fusarium* after exogenous ABA application, gene expression was significantly down-regulated in *PR2* (*P* < 0.01), *PR3* (*P* < 0.01), *PR4* (*P* < 0.001) and significantly up-regulated in *HPL* (*P* < 0.001), in comparison to the plant colonized by *Fusarium* without ABA application. In the plant co-colonized by *Metarhizium* and *Fusarium*, the expression of *PR1* (*P* < 0.01) and *PR3* (*P* < 0.01) was significantly down-regulated and the expression of *HPL* (*P* < 0.01) was significantly up-regulated after ABA application compared to the plant colonized by both fungi without ABA application. The application of ABA altered the expression of *ERF1*, *PR1* and *HPL* in un-inoculated control plant compared to the un-inoculated bean plant without ABA application, showing significant up-regulation of *ERF1* (*P* < 0.05) and *HPL* (*P* < 0.001) and significant down-regulation of *PR1* (*P* < 0.01).

### SAI after exogenous ABA application

SAI in bean leaves decreased significantly in the un-inoculated control plant and all fungi-treated group after ABA application when compared to the corresponding plant without ABA application (Supplementary Fig. S3).

## Discussion

**Figure 5:**
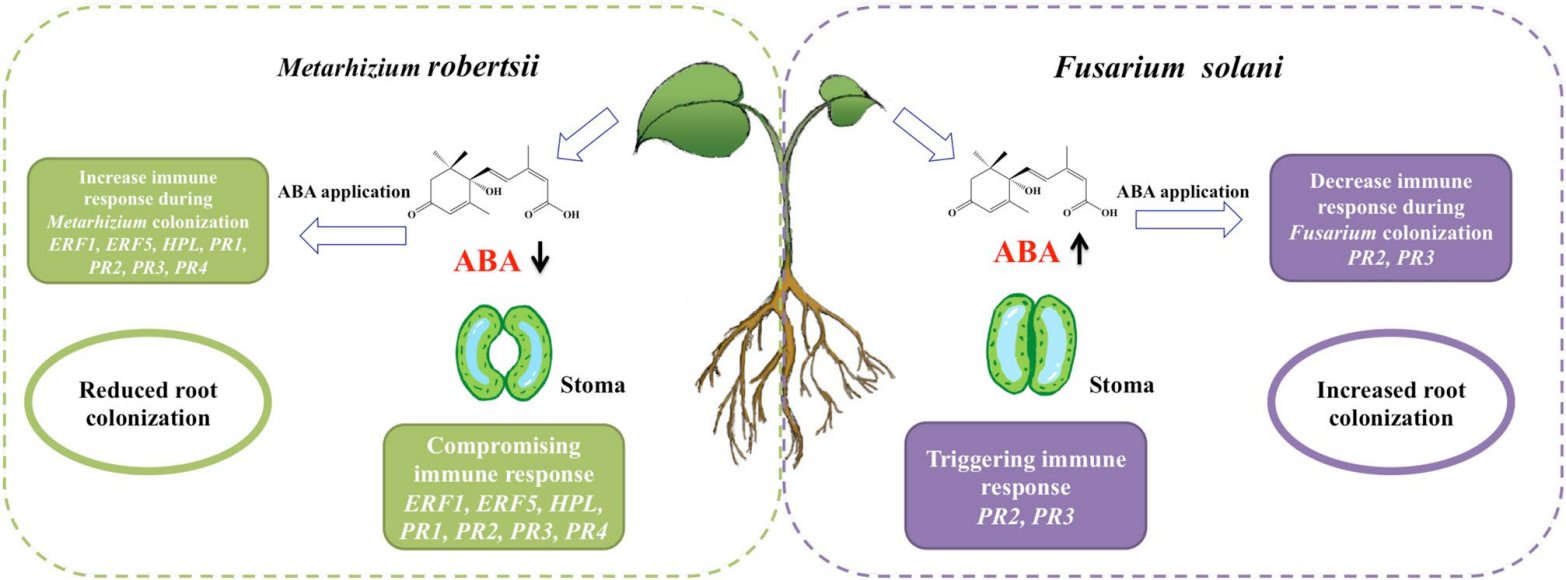
A schematic diagram depicting the differential plant immune responses during colonization by *M. robertsii* and *F. solani*. The left part of the diagram shows immune response of bean plant to colonization by *M. robertsii*. This endophytic colonization resulted in a reduction in abscisic acid (ABA), increased stomatal size and decreased relative expression of immune response genes in bean plant. The exogenous application of ABA decreased root colonization of *M. robertsii* and triggered the expression of immune response genes, which confirmed the role of ABA during endophytic colonization. The right part of the diagram shows immune responses of bean plant to colonization by *F. solani*. This pathogenic colonization resulted in an increase of ABA, reduced stomatal size and triggered the expression of immune response genes. This response was opposite to that found in endophytic colonization by *M. robertsii*. Exogenous ABA application increased root colonization by *F. solani* and repressed the expression of immune response genes, which suggests an important role of ABA modulation during endophytic colonization or pathogenic colonization. Pathogenesis related protein 1 (*PR1*), Beta 1–3 endoglucanase (*PR2*), Chitinase class I (*PR3*), Pathogenesis related protein 4 (*PR4*), Ethylene-Responsive Transcription Factor 1 (*ERF1*), Ethylene-Responsive Transcription factor 5 (*ERF5*), and Hydroperoxide lyase (*HPL*).

Phytopathogens, by definition, damage the plant during colonization; on the other hand endophytes can live in the plant asymptomatically. How the plant differentiates and allows colonization by endophytes, but mounts an immune response to phytopathogens, is still largely unknown. Our model system allows for an investigation into the differential plant immune responses to endophytic and pathogenic fungal colonization. *Metarhizium* is an insect biocontrol agent^23,24^ as well as an agricultural biofertilizer^21^. Understanding plant immune responses to this fungus can have promising implications in agricultural applications such as improving beneficial colonization by endophytes and resisting detrimental colonization by phytopathogens in crops^25^. Here we applied *M. robertsii*, an endophytic insect-pathogenic fungus to bean root. This fungus is also an antagonist of *Fusarium solani* f. sp. *phaseoli,* a pathogenic fungus that causes *Fusarium* root rot^4^. Our findings showed that the bean plant had relatively reduced ABA as well as larger stomatal openings during endophytic colonization by *Metarhizium.* Alternatively, increased ABA levels and smaller stomatal openings, and the triggering of general immune response genes were observed during a pathogenic response to *Fusarium* (Fig. 5). The exogenous application of ABA differentially influenced root colonization, transcriptional expression of catabolic genes of ABA and plant immune response genes in bean colonized by *Metarhizium* and *Fusarium* respectively, which confirmed the different roles of ABA during endophytic and pathogenic colonization (Fig. 5).

Bean plant concentrations of ABA, PA, and DPA that we observed were similar to that observed in unwilted bean leaves, which was much lower than that in wilted leaves^26–28^. Based on our results of the total amounts of ABA metabolites (Fig. 1A), we calculated that each bean plant contained ca. 4 μg, 3 μg and 5 μg of ABA metabolites respectively in the un-inoculated control, *Metarhizium* colonized and *Fusarium* colonized plant, respectively. Therefore, the amounts of 1.5, 7.5 and 20 μg ABA which we experimentally applied to the bean plant bracket the range of ABA levels found in bean plant.

ABA can control stomatal aperture during plant response to abiotic and biotic stresses^13^. Increased levels of ABA in the guard cells can induce stomatal closure and an increase in stomata size was observed in an ABA deficient mutant of *Arabidopsis*^29^. Increased stomatal openings during colonization by *Metarhizium* and decreased stomatal openings during colonization by *Fusarium* are consistent with ABA levels in bean leaves during fungal colonization (Fig. 1). At early stages of *Fusarium* infection, stomatal closure was mainly responsible for decreased CO_2_ assimilation which was attributed to leaf water deficit^30^. *Metarhizium* has been reported to translocate nitrogen directly from infected insect cadavers to the plant host in exchange for photosynthate^3,21^. As an entryway for CO_2_, the opening of stomata during colonization by *Metarhizium* may facilitate photosynthesis by improving intake of carbon dioxide, which may, in turn, benefit the fungus with more carbohydrate supplied from the plant. On the other hand, many pathogens can exploit stomata and cause them to open which facilitates entry, and indicates a passive process as stomatal opening is a sign of a compromised plant immune response^31^. The endophytic colonization by *Metarhizium* was observed through foliar application in soybean^32^, which indicated that opening of stomata during root colonization may also facilitate the subsequent entry of *Metarhizium* through the surface of bean leaves.

*PvCYP707As* which functions in the hydroxylation of ABA, plays a major regulatory role in controlling the level of ABA in plants, which can be transcriptionally induced in response to rehydration, submergence and developmental processes^26^. During plant infection, the up-regulation of *CYP707A3* was reported as a defense response by *Arabidopsis* against *B. cinerea*^33^. In our study, the up-regulation of *PvCYP707A1* and *PvCYP707A3* was observed in bean leaves during colonization by *F. solani* or *M. robertsii* in comparison to the un-inoculated plant.

Application of ABA can induce the expression of *CYP707As* in bean, maize, *Arabidopsis* and potato^26,34–36^, which is consistent with our observations of increased expression of *PvCYP707A1* and *PvCYP707A3* in bean leaves after exogenous application of ABA in the un-inoculated and *Metarhizium* colonized bean plant compared to plants without exogenous ABA application. Exogenous ABA application resulted in decreased expression of *PvCYP707A1* and *PvCYP707A3* in leaves during colonization by *Fusarium* compared to bean colonized by *Fusarium* without ABA application. The expression of ABA 8′-hydroxylase was also triggered by the increasing concentrations of NADPH and oxygen^36^ and inhibited by osmotic stresses and phytochrome-dependent signaling pathways^37,38^. This down-regulation of *PvCYP707As* after ABA application in *Fusarium* colonized bean may indicate other factors are involved in the regulation of ABA catabolism in bean plant under the disease state during exogenous ABA application. Endogenous ABA content may be maintained by a balance between biosynthetic and catabolic activities. ABA biosynthesis was reported by a representative enzyme nine-*cis* epoxycarotenoid dioxygenase (NCEDs)^28^, which showed no significant differences in the un-inoculated bean plant and fungal colonized bean plant (Supplementary Fig. S4). This result indicated that there may be other enzymes or factors in the ABA biosynthetic pathway, which might be influenced by fungal colonization^39^. Research needs to be directed at target key enzymes or fungal effectors that can affect the ABA biosynthetic and catabolic pathways during endophytic colonization and/or pathogenic colonization.

Pathogenesis related proteins play a fundamental role in compromising the cell wall of the fungal pathogen and are downstream components of systemic acquired resistance^40^. Transcriptional profiles showed the up-regulation of *PR* genes after fungal infection in agricultural plants^22^. Meanwhile the overexpression of *PR* genes in plants leads to increased resistance to biotrophic and necrotrophic fungal phytopathogens^40^. The down-regulation of *PR1*, *PR2*, *PR3* and *PR4* indicated a compromised immune response to endophytic colonization by *Metarhizium* in bean plant. Thus, the cell wall of *M. robertsii* may avoid recognition and subsequent attack from plant degradative enzymes, thereby bypassing the plant immune system. A down-regulation of pathogenesis related genes has been reported in peanut roots when colonized by *M. anisopliae*^6^, which may indicate that a similar plant immune response of pathogenesis related proteins may be shared during endophytic colonization by *Metarhizium*. On the other hand, plant colonization by *Fusarium* triggered the expression of *PR2* and *PR3*, which results in a plant response to this phytopathogen. Exogenous ABA application induced transcriptional expression of *PR2*, *PR3*, and *PR4* in plants colonized by *Metarhizium* whereas these genes were repressed in the plant colonized by *Fusarium* compared to the corresponding fungi colonized plant without ABA application. The differential expressions of *PR*s may result in decreased root colonization by *Metarhizium* and increased colonization by *Fusarium* after the exogenous application of ABA compared to the control without ABA application. ABA is involved in differential expression of *PR*s during the endophytic colonization and pathogenic colonization. *PR1* can be repressed by exogenous ABA in the un-inoculated bean. A similar situation has been reported in tobacco cell cultures where ABA can inhibit the transcription of a beta 1–3 glucanase^41^. The complex effects of ABA on the transcriptional expression of *PR*s have been reported during different pathogenic processes. *PR2* was negatively regulated by ABA in response to the hemibiotrophic fungus *Leptosphaeria maculans* in *Arabidopsis*^42^. While the expression of *PR4* and *PR1* family members was induced by exogenous ABA application in healthy wheat as well as wheat infected by *F. graminearum*^43^.

The ethylene-responsive transcription factors can regulate transcription of defense genes by integrating signals from the ethylene and JA pathways during induced systemic resistance^44^. The overexpression of *ERF1* show selective resistance to different fungi and bacteria in *Arabidopsis,* which enhanced resistance to necrotrophic pathogens such as *B. cinerea*, *F. oxysporum*, and *Plectospherella cucumerina*, and reduced tolerance to the hemibiotrophic bacteria *Pseudomonas syringae* pv. *tomato*^45^. Constitutive expression of *ERF5* resulted in significantly increased resistance against *B. cinerea* in *Arabidopsis*^46^. The expression of *ERF*s can be induced by ABA in cotton, tobacco and tomato^47–49^. We have also observed the up-regulation of *ERF1* after exogenous ABA application in un-inoculated bean. Meanwhile, the exogenous application of ABA significantly increased expression of *ERF1* and *ERF5* during colonization by *M. robertsii*. However, no significant differences in the expression of *ERF1* and *ERF5* were observed during colonization by *F. solani*, which indicated these two genes are not involved in the early stage of plant immune responses to the pathogenic colonization by *F. solani*.

The significant down-regulation of *PR2*, *PR3* and *PR4* in bean co-colonized by *M. robertsii* and *F. solani* compared to plant colonized only by *F. solani* or *M. robertsii* may indicate that these genes play a profound role in the interactions between *M. robertsii* and *F. solani* in the bean plant at the early stages of colonization. Our results expand upon the potential mechanisms of *Fusarium* disease suppression by *Metarhizium* within the plant, besides the direct antagonistic effect of *Metarhizium* on *Fusarium*^4^. These mechanisms include the indirect effects on the regulation of gene expression involved in immune defense responses in the plant host during endophytic and pathogenic colonization.

In conclusion, the present work suggests that ABA plays a central role in reducing immune responses during endophytic colonization by *M. robertsii* and inducing immune responses to pathogenic colonization by *F. solani* during the early stages of fungal infection in bean plants. Decreased fungal root colonization together with the increased expression of plant immune response genes *PR1*, *PR2*, *PR3*, *PR4*, *EFR1*, *ERF5* and *HPL* in *M. robertsii*-colonized bean plant after exogenous application of ABA indicated that ABA treatment can increase plant resistance to the endophytic colonization by *M. robertsii*. However, increased root colonization of *F. solani* and reduced expression of *PR2* and *PR3* in *F. solani*-colonized bean after ABA treatment suggested that the exogenous application of ABA could increase plant susceptibility to pathogenic colonization by *F. solani*. This information can potentially be utilized in agriculture to increase beneficial colonization by endophytes and also increase plant protection from phytopathogens through the modification of ABA pathways, or the alteration of ABA, in agricultural plants.

## Materials and methods

### Fungal culture

*M. robertsii* ARSEF 2575-GFP (*M. robertsii*) expressed green fluorescent protein (GFP)^50^. *Fusarium solani* f. sp. *phaseoli* (DAOM170970) was obtained from the Canadian Culture Collections, Agriculture Canada, Ottawa, which was initially isolated from infected bean roots. *F. solani* and *M. robertsii* were grown at 27 °C on potato dextrose agar (PDA; Difco Laboratories, BD, Mississauga, Ontario, Canada). Conidia of these fungi were dislodged from a 12 day old agar culture with 0.01% Triton X-100. To enrich for conidia, the slurry was passed through a funnel containing glass wool. The concentration of the suspension was adjusted to 10^6^ conidia mL^−1^ using a hemocytometer for counting.

### Plant materials and growth condition

Seeds of *Phaseolus vulgaris* were obtained from OSC Seeds, Waterloo, Ontario, Canada. The seeds were surface sterilized in order to prevent fungal or bacterial contamination. Seeds were immersed in sterile distilled water for 15 min and subsequently immersed in 4% sodium hypochlorite solution three times for 5 min. Each time, after decanting the fluid, the seeds were rinsed with sterile distilled water. Axenically treated seeds were kept at 4 °C overnight to allow for synchronization of growth and then placed onto the 1.5% water agar medium with a photoperiod of 16 h at 25 °C. The sterilized seeds were tested for contamination by fungi or bacteria by plating onto PDA. Beans developed visible roots in 5 days on 1.5% water agar.

Soil (Schultz Potting Mixture, Brantford, ON, Canada) was sterilized by autoclaving at 121 °C for 20 min three times. Plants were grown in plastic garden pots (10 cm in height by 15 cm in diameter). The garden pots were sterilized with UV light in biosafety cabinet for 3 h prior to use. The pots were filled with sterile soil to 9 cm from the bottom, and moistened with sterile distilled water. Each pot was planted with one sterile germinated soldier bean seed. A modified soil drench method was used to inoculate the soil with fungal conidia suspension^51^. The seedlings were grown in the presence or absence of the conidial suspension in four conditions: (1) 5 mL of 10^6^ conidia ml^−1^ suspension of *M. robertsii* (M); (2) 5 mL of 10^6^ conidia ml^−1^ suspension of *F. solani* (F); (3) 5 mL of 10^6^ conidia ml^−1^ suspension of *M. robertsii* together with 5 mL of 10^6^ conidia ml^−1^ suspension of *F. solani* (FM); (4) 5 mL 0.01% Triton X-100 as the control. The fungal suspension was pipetted onto the surface of the soil surrounding the seedling. The pots were then kept at 25 °C for a photoperiod of 16 h a day with 70% humidity in the growth chamber. Light in the chamber was provided by fluorescent tubes (Sylvania 17 W T8 4100 K). Plants were watered daily with sterile distilled water.

The first bean leaves with no visible disease symptoms were collected at day 14 post inoculation, quickly frozen in liquid nitrogen and then stored in a deep freezer (− 80 °C) until lyophilization.

### Plant hormone analysis

The quantification of ABA, ABAGE, DPA, PA, 7′-hydroxy-abscisic acid, *neo*-phaseic acid, *trans*-abscisic acid, cytokinins, auxins, gibberellins and 1-aminocyclopropane-1-carboxylic acid (ACC) in bean foliar tissue was performed using an ultra-performance liquid chromatography-electrospray tandem mass spectrometry (UPLC-ESI–MS/MS) at the National Research Council of Canada-Saskatoon^52,53^. The analyses utilizes the multiple reaction monitoring (MRM) function of the MassLynx v4.1 (Waters Inc) control software. The resulting chromatographic traces were quantified off-line by the QuanLynx v4.1 software (Waters Inc). Each trace was integrated and the resulting ratio of signals (non-deuterated/internal standard) was compared with a previously constructed calibration curve to yield the amount of analyte present (ng per sample). Calibration curves were generated from the MRM signals obtained from standard solutions based on the ratio of the chromatographic peak area for each analyte to that of the corresponding internal standard. The quality control (QC) samples, internal standard blanks and solvent blanks were also prepared and analyzed along each batch of tissue samples. The experiments were done with 2 replicates. Each replicate pooled 6 biological samples with 2 experimental replicates (3 biological replicates for each experimental replicates) in order to assess plant hormones for each group. The plant hormones were quantified in nanograms per gram of dry weight of bean foliar tissue (ng g^−1^ DW).

### Stomatal size assays

Two weeks old bean plants grown under the same conditions described above for the quantification of plant hormones were used for measuring stomatal aperture index (SAI) with modified protocols^20^. Briefly, clear nail polish was applied to the abaxial surface of the first true leaves. When the polish was dry, it was peeled with transparent tape (Duramax Inc.) and attached to a glass slide. The width and length of the stomatal apertures in the stomatal impression were measured using an ocular meter with a Leitz Diaplan light microscope at 1000× magnifications. SAI was calculated as the division of the stomatal aperture width by the length. Stomata (N = 63) of each stomatal impression were examined with 14–16 leaf impressions in each treatment. Red fluorescence microscopy was performed by dying the abaxial surface of the leaves with 1 μM rhodamine 6G (Sigma)^20^. The images were taken under a Leitz Diaplan microscope equipped with light filters (TXRED-4040B; excitation filter: 562 nm, dichroic mirror: 593 nm, emission filter: 624 nm). All images were captured with a Leica DFC-400, Microsystems using Leica Application Suite V3 software.

### ABA application experiment

To confirm the role of ABA during the endophytic colonization by *M. robertsii* and pathogenic colonization by *F. solani*, ABA was applied to the bean plant and root colonization by fungi was assessed. A modified ABA application method was used on each bean plant^54^. Briefly, doses of ABA 1.5 μg, 7.5 μg, and 20 μg in 10 μL of 25% aqueous ethanol were placed in the nodes of first true leaf on each plant at day 7 after placement in the soil. The 10 μL drop was delivered using a 26G 1/2 needle (BD, Franklin Lakes, NJ) and allowed to sit on the wounded node until it had been absorbed. Compared to the control plant with 10 μL 25% aqueous ethanol, the fungal colonized plant without ABA application showed no significant differences in the fungal root colonization (Supplementary Fig. S5), which suggests that the application of ABA did not affect root colonization by fungi in bean plant.

To further confirm the influence of ABA itself on fungi in vitro, the morphology of *M. robertsii* and *F. solani* on PDA plates grown in the presence of ABA. A conidial suspension ( 5 μL of a 10^6^ conidia/mL in 0.01% Triton X-100 suspension) was inoculating into the center of PDA plates (10 cm Petri dish with 10 mL potato dextrose agar medium) amended with 20 μg, 100 μg in 10 μL of 25% aqueous ethanol. The plates were incubated at 27 °C. The colony morphology of *M. robertsii* and *F. solani* was examined at day 14. The conidial yields of *M. robertsii* and *F. solani* were assessed^55^. Briefly, a 7 mm diameter plug was taken half way between the center of colony to the edge of colony at day 14. The plug was immersed in 1.0 mL 0.01% Triton X-100 and vortexed to count the conidia concentration via hemocytometer.

### Root colonization experiment

Plants were harvested at day 14 and thoroughly washed until any remaining soil was removed from the roots. The plant roots with no visible disease symptoms were weighed and then homogenized using a rotary homogenizer (Greiner Scientific, Frickenhausen Germany) in 0.01% Triton X-100. After vortexing, two hundred microliters of this homogenate, in duplicate, was then spread onto a Petri dish containing media. For quantification of *M. robertsii*, potato dextrose agar supplemented with 1 g L^−1^ yeast extract, 0.5 g L^−1^ chloramphenicol, 0.25 g L^−1^ cycloheximide and 4 mg L^−1^ thiabendazole^56^ was used and CFU were counted. Colonies of *M. robertsii* were further confirmed by visualization of GFP fluorescence. To quantify CFU of *F. solani*, Malachite Green Agar 2.5 ppm (MGA 2.5) selective medium was used (15 g L^−1^ peptone, 1.0 g L^−1^ KH_2_PO_4_, 0.50 g L^−1^ MgSO_4_ 7H_2_O, 15 g L^−1^ agar, 0.50 g L^−1^ chloramphenicol, 0.30 g L^−1^ streptomycin sulfate salt, and 2.5 mg L^−1^ malachite green oxalate)^57^. The CFU were counted on day 4 and confirmed on day 12.

### Reverse-transcribed quantitative PCR (RT-qPCR)

Total RNA was prepared from the first bean leaves and roots under the same conditions described above for the quantification of plant hormones at day 14 for the expression of *PvCYP707A1* (ABA 8’-hydroxylase A1) and *PvCYP707A3* (ABA 8′-hydroxylase A3)^26^. The bean leaves and roots were separately frozen in liquid nitrogen and ground to fine power using a mortar and pestle. The QIAzol Lysis Reagent (Qiagen) was used for RNA extraction, and all samples were DNase-treated using RNase-Free DNase (Promega). The RNA concentration was determined spectrophotometrically using Qubit (Invitrogen). Total RNA was transcribed into complementary DNA (cDNA) with High Capacity cDNA reverse transcription kit (Applied Biosystems, Thermofisher Scientific), following manufacturer’s instructions. Real-time PCR was conducted using a SensiFAST™ SYBR No-ROX kit (Bioline) in a volume of 10 μl, including 5 μl 2 × SensiFAST SYBR® No-ROX Mix, 2 µl cDNA, 0.5 µl of each forward and reverse primers (5.0 µM). The PCR protocol included a 2 min initial denaturation step at 95 °C, followed by 40 cycles of 5 s at 95 °C and 30 s at 63 or 68 °C. Fluorescence measurements were collected at each polymerization step then held at 95 °C for 10 s. The melting curve (65–95 °C) was taken at 0.5 °C intervals. PCR products were checked with melt curve analysis after quantification. Primers are listed in Supplementary Table S2. The expression of *PR1*, *PR2, PR3*, *PR4*, *ERF1*, *ERF5,* and *HPL* was tested on bean leaves^58^. The relative expression levels of each gene were normalized against the reference gene, *actin*^59^ by Bio-Rad CFX Manager software. After gradient PCR with annealing temperatures from 55 to 68 °C, 63 °C was chosen for annealing temperature to determine the transcript levels of *ERF1*, *PR1*, *PR2*, *PR3*, and *PvCYP707A1* and 68 °C was chosen for the transcript levels of *ERF5*, *HPL*, *PR4* and *PvCYP707A3*. These conditions excluded amplification of DNA from *M. robertsii* or *F. solani*. Four biological replicates with two technical replicates were performed independently for each gene tested.

## Supplementary Information

Supplementary Information.

## Data Availability

All data generated or analysed during this study are included in this published article (and its Supplementary Information files).
