# Supplementary material for: Abscisic acid implicated in differential plant responses of Phaseolus vulgaris during endophytic colonization by Metarhizium and pathogenic colonization by Fusarium
Source: Sci Rep. 2021 May 31;11:11327. doi: 10.1038/s41598-021-90232-4 (PMC8167117; doi:10.1038/s41598-021-90232-4)
Supplement: Supplementary file 1 — Supplementary Information. [file 41598_2021_90232_MOESM1_ESM.pdf]

Abscisic acid implicated in differential plant responses of *Phaseolus vulgaris* during endophytic colonization by *Metarhizium* and pathogenic colonization by *Fusarium*

Shasha Hu and Michael J. Bidochka*

Department of Biological Sciences, Brock University, St. Catharines, ON Canada L2S 3A1

*Address correspondence to

Email: mbidochka@brocku.ca

Telephone: +1 905-688-5550

Supplementary Information

Supplementary Figure S1. The concentration of important plant hormones during endophytic or pathogenic colonization.

Supplementary Figure S2. Colony morphology and conidial production of *M. robertsii* and *F. solani* under different amount of ABA.

Supplementary Figure S3. Analysis of stomatal aperture during the exogenous application of ABA on bean leaves.

Supplementary Figure S4. Relative expression of genes involved in ABA biosynthetic pathway in bean leaves and roots.

Supplementary Figure S5. CFU of *M. robertsii* or *F. solani* in bean root homogenate after exogenous application of 25% ethanol.

Supplementary Table S1. Plant hormones tested in this study

Supplementary Table S2. Primer sequences of *Phaseolus vulgaris* used for RT-PCR analysis.

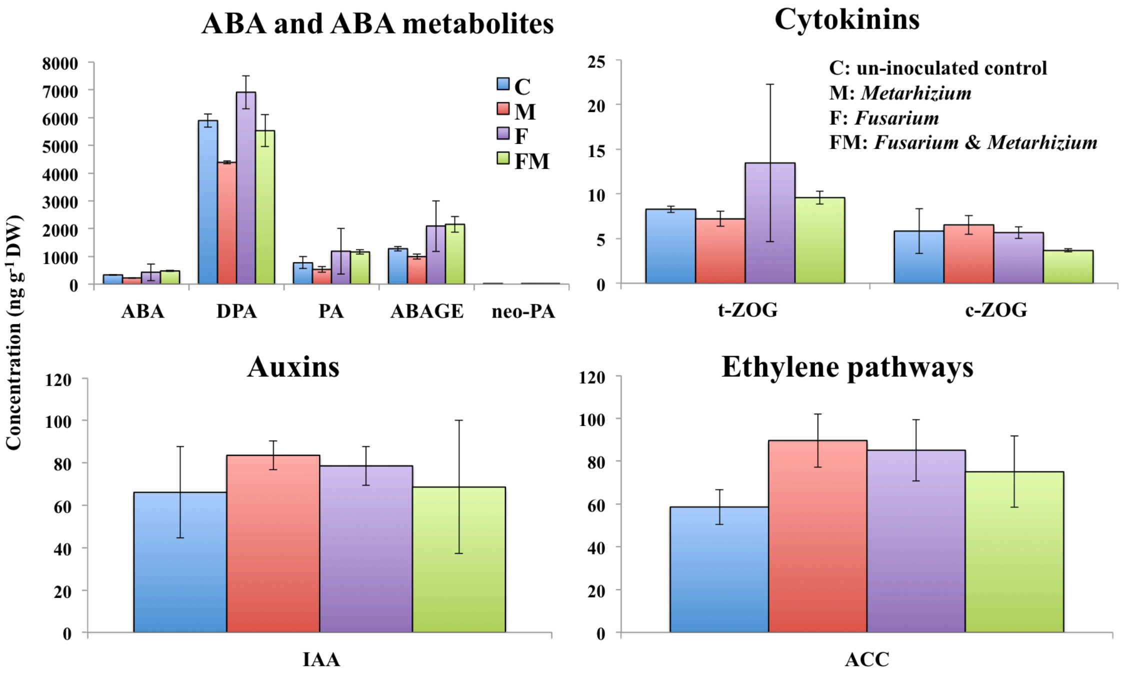

Supplementary Figure S1. The concentration of important plant hormones during endophytic or pathogenic colonization. *cis*-Abscisic acid (ABA), Dihydrophaseic acid (DPA), Phaseic acid (PA), Abscisic acid glucose ester (ABAGE), neo-PA (neo-Phaseic acid), (*trans*) zeatin-O-glucoside (*t*-ZOG), (*cis*) zeatin-O-glucoside (*c*-ZOG), indole-3-acetic acid (IAA), 1-aminocyclopropane-1-carboxylic acid (ACC).

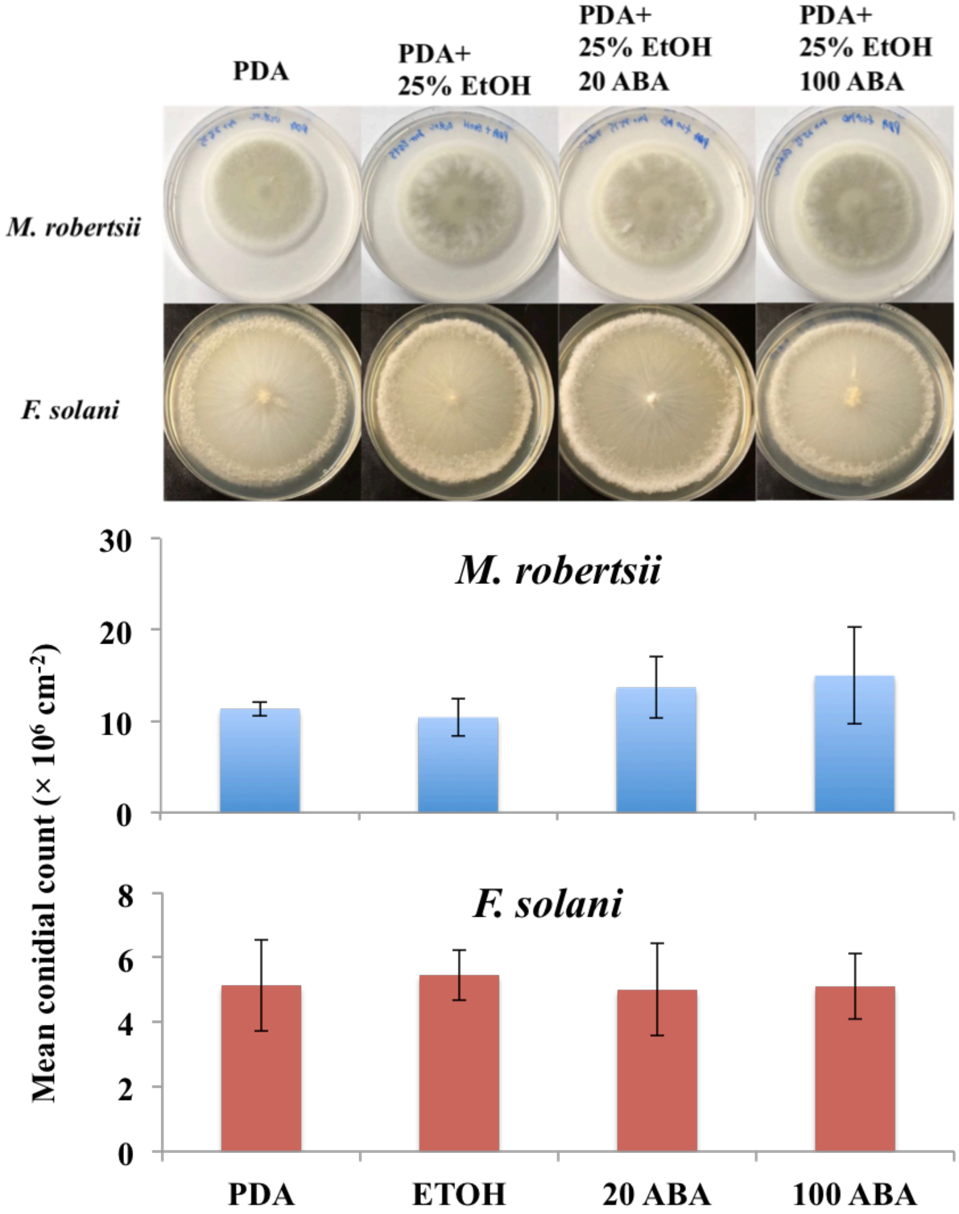

Supplementary Figure S2. Colony morphology and conidial production of *M. robertsii* and *F. solani* under different amount of ABA.

ABA, abscisic acid. X-axis represents values of ABA μg per PDA plate.

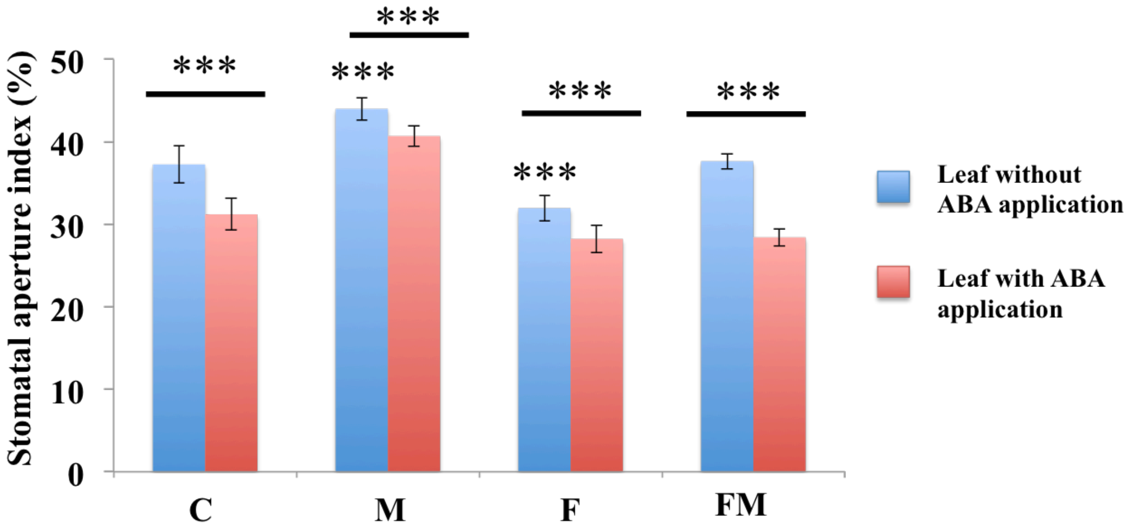

Supplementary Figure S3. Analysis of stomatal aperture during the exogenous application of ABA on bean leaves. The stomatal aperture index in the bean leaves after 14 days of un-inoculated control (C) and bean colonized by *M. robertsii* (M), *F. solani* (F), and *M. robertsii* together with *F. solani* (FM). Standard deviations are shown. Data were analyzed by one-way ANOVA with Fisher's least-significant difference (LSD) test. Statistical differences shown; * $P < 0.05$; ** $P < 0.01$; *** $P < 0.001$. Asterisks alone indicate statistically significant differences relative to un-inoculated control. Asterisk above black line indicate the significant differences of stomatal aperture index with exogenous ABA application compared to that in corresponding plant without ABA application.

Gene Expression in Bean Leaves

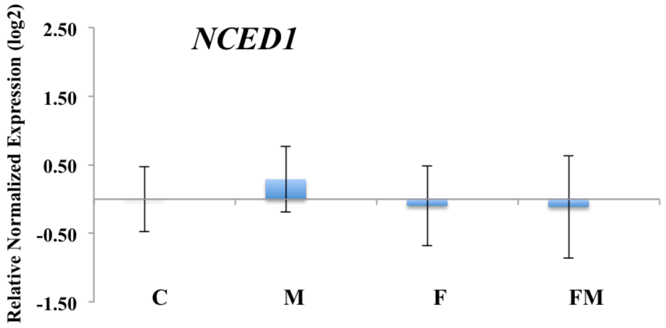

Gene Expression in Bean Roots

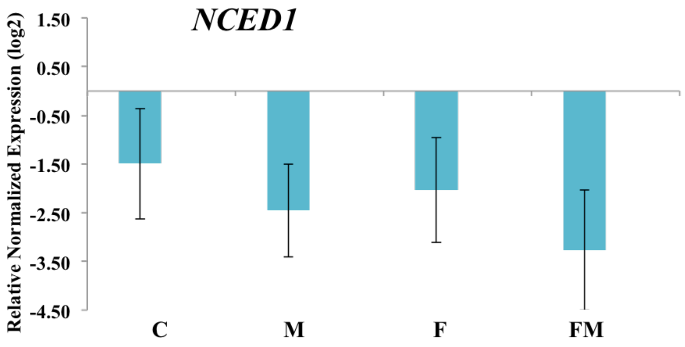

Supplementary Figure S4. Relative expression of genes involved in ABA biosynthetic pathway in bean leaves and roots. RNA was extracted at 14 days bean leaf and root in un-inoculated control (C), colonized by *M. robertsii* (M), *F. solani* (F), and *M. robertsii* together with *F. solani* (FM). For each gene, the expression level in un-inoculated control bean leave was set to 1. Standard errors are shown. Figure Data were analyzed with standard t-test in Bio-Rad CFX Manager software. No statistical differences are shown nine-*cis* epoxycarotenoid dioxygenases 1(NCED1).

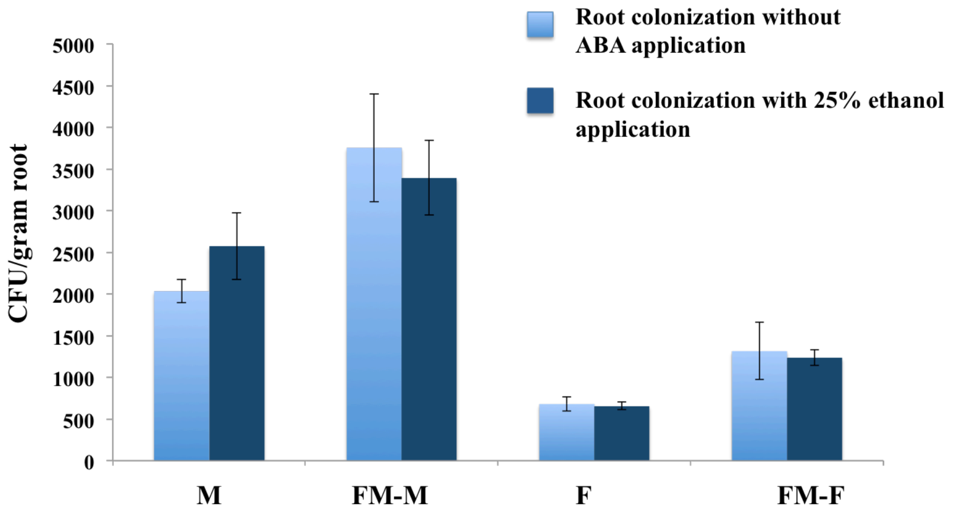

Supplementary Figure S5. CFU of *M. robertsii* or *F. solani* in bean root homogenate after exogenous application of 25% ethanol. M: *M. robertsii*, F: *F. solani*, FM: *F. solani* and *M. robertsii* treatment group. Standard error bars are shown.

Supplementary Table S1 Plant hormones tested in this study

Group	Abbreviation	Full Name
**ABA and ABA metabolites**	ABA	*cis*-Abscisic acid
	ABAGE	Abscisic acid glucose ester
	DPA	Dihydrophaseic acid
	PA	Phaseic acid
	7'OH-ABA	7'-Hydroxy-abscisic acid
	neo-PA	*neo*-Phaseic acid
	t-ABA	*trans*-Abscisic acid
**Auxins**	IAA	Indole-3-acetic acid
	IAA-Asp	N-(Indole-3-yl-acetyl)-aspartic acid
	IAA-Glu	N-(Indole-3-yl-acetyl)-glutamic acid
	IAA-Ala	N-(Indole-3-yl-acetyl)-alanine
	IAA-Leu	N-(Indole-3-yl-acetyl)-leucine
	IBA	Indole-3-butyric acid
**Cytokinins**	*t*-ZOG	(*trans*) Zeatin-O-glucoside
	c-ZOG	(*cis*) Zeatin-O-glucoside
	t-Z	(*trans*) Zeatin
	c-Z	(*cis*) Zeatin
	dhZ	Dihydrozeatin
	t-ZR	(*trans*) Zeatin riboside
	c-ZR	(*cis*) Zeatin riboside
	dhZR	Dihydrozeatin riboside
	iP	Isopentenyladenine
	iPR	Isopentenyladenosine
	KIN	Kinetin
**Ethylene**	ACC	1-aminocyclopropane-1-carboxylic acid
**Gibberellins**	GA1	Gibberellin 1
	GA3	Gibberellin 3
	GA4	Gibberellin 4
	GA7	Gibberellin 7
	GA8	Gibberellin 8
	GA9	Gibberellin 9
	GA19	Gibberellin 19
	GA20	Gibberellin 20
	GA24	Gibberellin 24
	GA29	Gibberellin 29
	GA34	Gibberellin 34
	GA44	Gibberellin 44
	GA51	Gibberellin 51
	GA53	Gibberellin 53

Supplementary Table S2 Primers sequences of *Phaseolus vulgaris* used for RT-PCR analysis

Gene	NCBI Sequence ID	Size (bp)	Forward Sequence	Reverse Sequence
Act	XM_007162263	126	CACCGAGGCACCGCTTAATC	CGGCCACTAGCGTAAAGGGAA
ERF1	XM_007144028	161	CGCTCTCAAGAGGAAACACTCC	TGAATCAGAAGGAGGAGGGAAT
ERF5	XM_007157197	151	GGCTCCAAGTGGATTGAGAAC	TCAGAATCAGATAACTACAAAGCACAA
HPL	XM_007149930	153	TCAAGGCTACATTTGTATTTCCA	TGGTGCACATTTCTTAGTAGCAA
PR1	XM_007154263	98	TGGTCCTAACGGAGGATCAC	TGGCTTTTCCAGCTTTGAGT
PR2	XM_007154264	246	GTGAAGGACGCCGATAACAT	ACTGAGTTTGGGGTCGATTG
PR3	XM_007137247	111	TGGAGTTGGTTATGGCAACAA	ATTCTGATGGGATGGCAGTGT
PR4	XM_007147114	140	CGCAGTGAGTGCATATTGCT	TGGTGCACATTTCTTAGTAGCAA
NCED1	AF190462	162	CTCCTTTTCTACGCTCGCAG	GGTGGTTAAGTCGCCGTTAG
PvCYP707A1	DQ352541	111	GCCTTTTGGCAGTGGGATTCAT	GCACCCACAACAGACCACCT
PvCYP707A3	DQ352543	121	AGACTACCACACTCGCCTCA	GCCATCACCCCAAGAGTTCA
